# Negative Pressure Therapy for Management of High Output Duodenal Fistula

**DOI:** 10.7759/cureus.9075

**Published:** 2020-07-08

**Authors:** Omar S El-Ghazzawy, Jordan Shively, Christian Massier

**Affiliations:** 1 General Surgery, Cleveland Clinic South Pointe Hospital, Warrensville Heights, USA; 2 General Surgery, South Pointe Hospital Cleveland Clinic Foundation, Beachwood, USA

**Keywords:** duodenal fistula, negative pressure wound therapy

## Abstract

This case study describes a patient with duodenal perforation caused by foreign body ingestion who postoperatively developed high output duodenal fistula and intra-abdominal sepsis. The management of this complication included the unique application of negative pressure wound vacuum therapy via retroperitoneal drains. This assisted the closure of the duodenal fistula, prevented surgical reintervention, helped control intra-abdominal sepsis, and simplified local wound care. Application of closed incision negative pressure therapy to the retroperitoneal space via surgical drains is a technically easy and well-tolerated modality that accelerates duodenal fistula closure.

## Introduction

Retroperitoneal infections secondary to duodenal and pancreatic leaks are devastating complications that can emerge from the repair of surgical duodenal injuries. These complications can further manifest into the development of a duodenal fistula. The management of a duodenal fistula is difficult due to the high volume output and caustic nature of upper gastrointestinal secretions. Duodenal fistula typically declares itself from postoperative day 5 to 10 and presents with bilious drainage associated with sepsis. Morbidities associated with the complication include wound infection, intra-abdominal infection, severe electrolyte abnormalities, and severe malnutrition [1]. Most duodenal fistulas are first managed with non-operative measures as operative intervention can be a highly morbid endeavor (30%) [2].

There have been case studies that describe various techniques of using negative pressure therapy for the management of duodenal fistula. These include endoluminal vacuum devices for the management of duodenal perforation [3] and closed wall suction to an open retroperitoneal wound [4]. Both of the aforementioned techniques of applying negative pressure to the retroperitoneal space have drawbacks for the patient. The endoluminal vacuum device subjects the patient to frequent endoscopies for changes of the wound vacuum sponge. The wound vacuum placed in the surgical wound is labor-intensive and painful to change for the patient. Our case presents the unique application of a wound vacuum device via retroperitoneal drains in the management of duodenal fistula and retroperitoneal infection, effectively representing a closed incision, skin level application of negative pressure therapy into the retroperitoneal space.

## Case presentation

**Figure 1:**
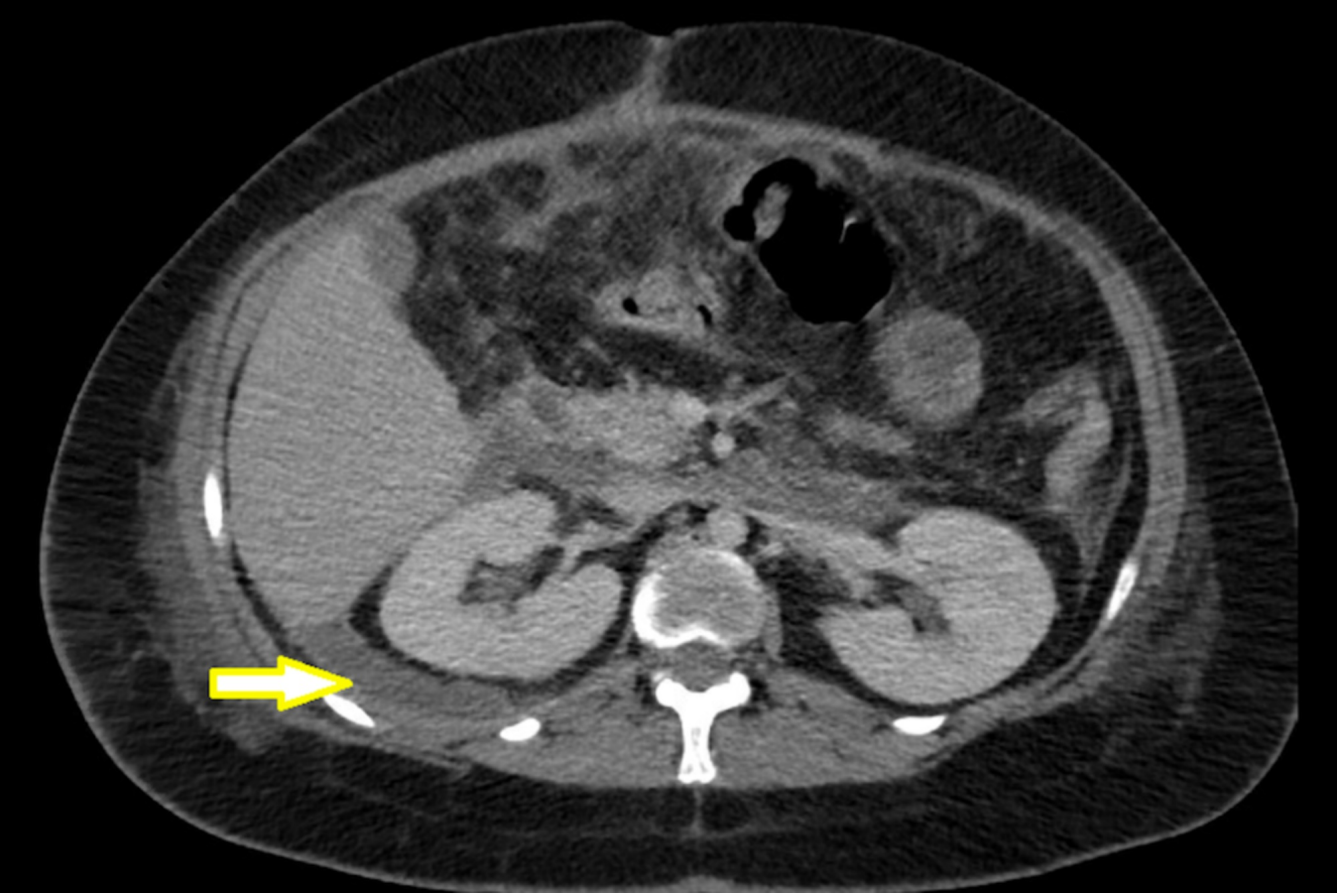
Axial image of right pararenal and retroperitoneal fluid collections

The case involves a 20-year-old female with extensive psychiatric issues and a history of swallowing foreign bodies on multiple occasions. She presented after swallowing multiple screws and underwent endoscopic retrieval of the screws. She later developed progressive abdominal pain, back pain, and fevers. A CT scan revealed a large retroperitoneal fluid collection secondary to duodenal perforation. She underwent exploratory laparotomy with washout of the retroperitoneal abscess, primary repair of duodenal perforation with pyloric exclusion, and gastrojejunostomy. Her postoperative course was complicated by the development of a duodenal fistula with high output, upwards of 1L per day. She was consequently made nil per oral and started on total parenteral nutrition. The patient was intermittently septic with persistent fevers. Repeat CT scan revealed peri-pancreatic and right peri-renal fluid collections (indicated by the arrow in Figure 1), despite the presence of two large-bore retroperitoneal drains.

**Figure 2:**
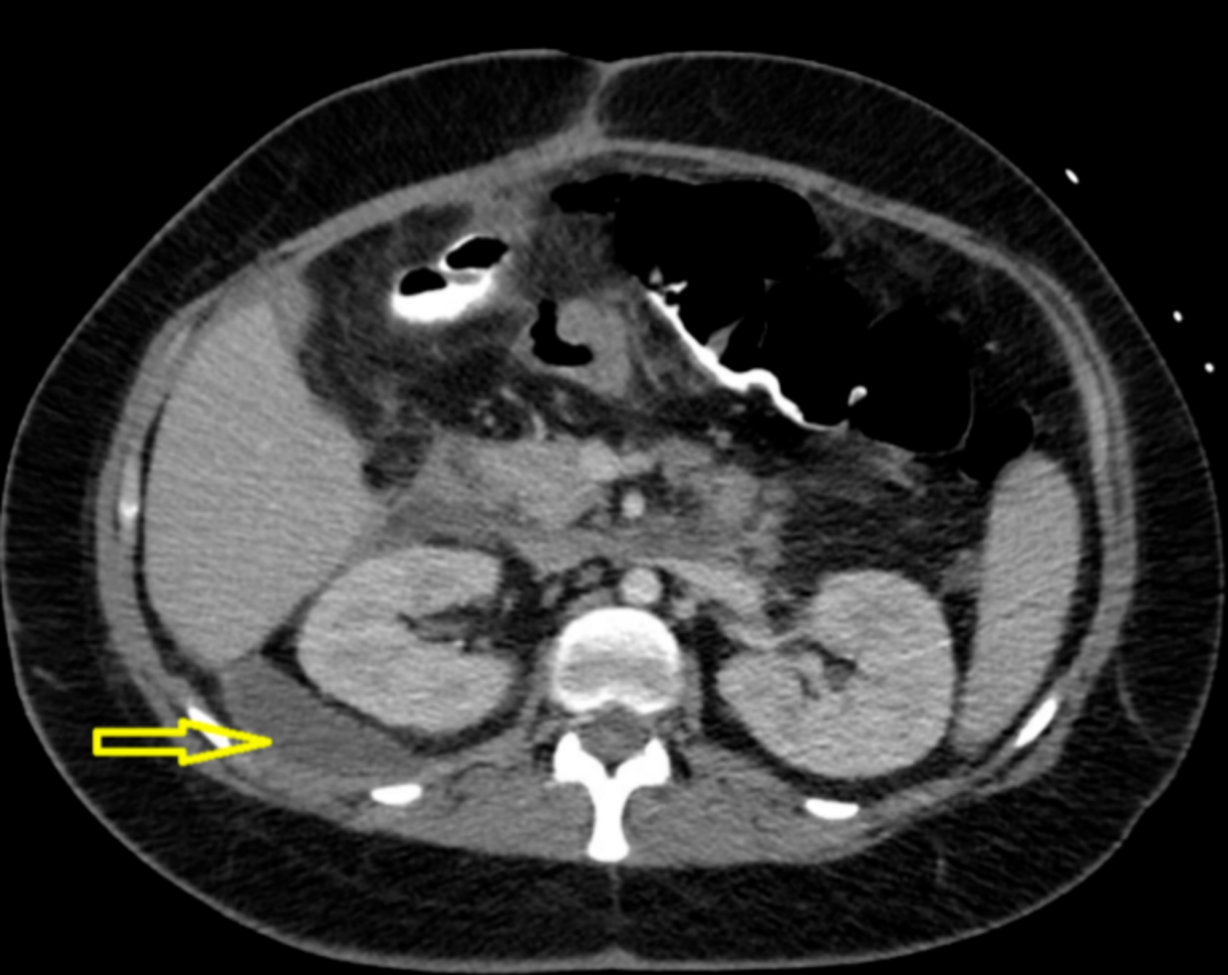
Axial image showing persistence of right para-renal fluid collection

Fevers occurred consistently at the same time at night, and it was determined that her intermittent sepsis was likely due to inadequate suction from the Jackson-Pratt bulbs. Multiple attempts were made to improve the drainage of the retroperitoneal abscesses. This included increasing the frequency of drain bulb emptying, increasing the size of the drain bulbs, and having the patient lay in the right lateral recumbent position to increase the dependency of drainage. Unfortunately, her intermittent sepsis persisted, and there was inadequate drainage of her fluid collections, as demonstrated by the follow-up CT scan (Figure 2).

**Figure 3:**
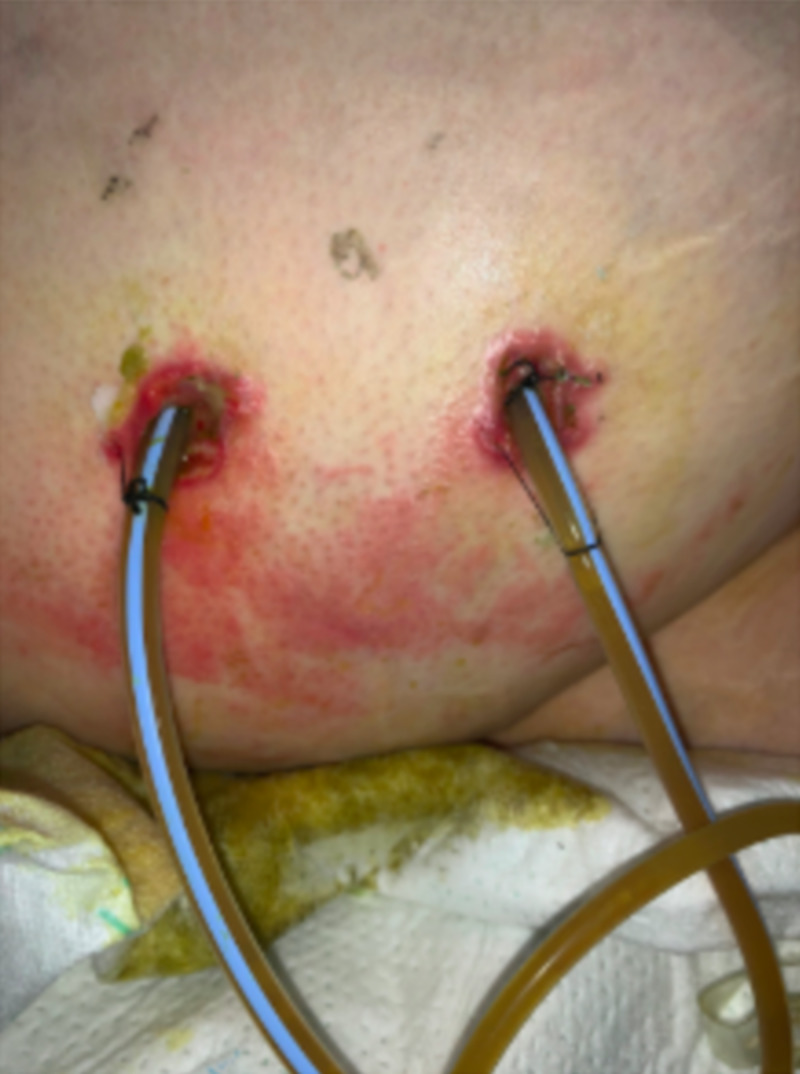
Lesions over the right lateral abdominal wall surrounding drain sites

Additionally, the patient was developing severe excoriated wounds to her skin around the drain sites from bile leakage (Figure 3).

**Figure 4:**
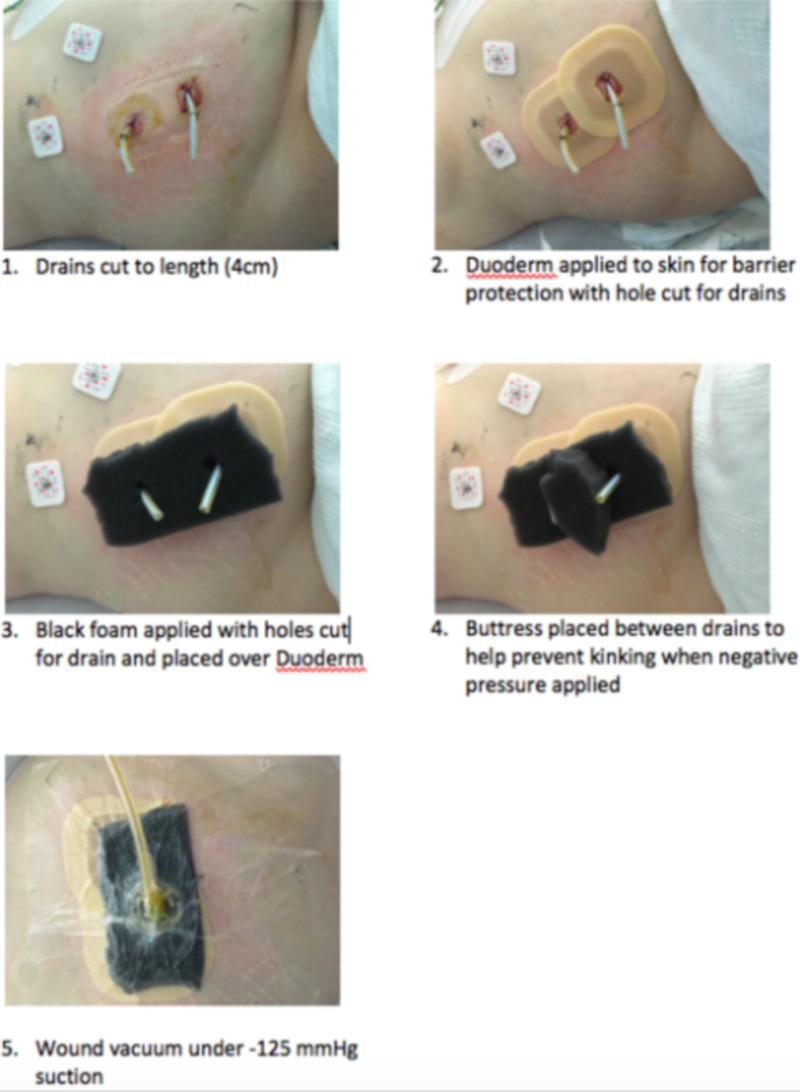
Application of negative pressure wound vacuum device to retroperitoneal drains

To combat this, we decided to place Duoderm® (Convatec, Skillman, NJ) and a wound vacuum device over the drain sites in order to provide continuous negative pressure to the retroperitoneum and improve local wound care. The numerated description of the technique is described below and in Figure 4:

1. Drains cut to length (4cm past the skin)

2. Duoderm applied to the skin with holes cut for drains as a skin barrier protection

3. Black foam applied with holes cut for drain and placed over Duoderm

4. Buttress placed between drains for support to help prevent kinking

5. Wound vacuum application applied with "lily pad" over the ends of drain

The negative pressure wound vacuum device was changed every three days. During each dressing change, the two retroperitoneal drains were withdrawn 2 cm each until complete removal in three weeks.

**Figure 5:**
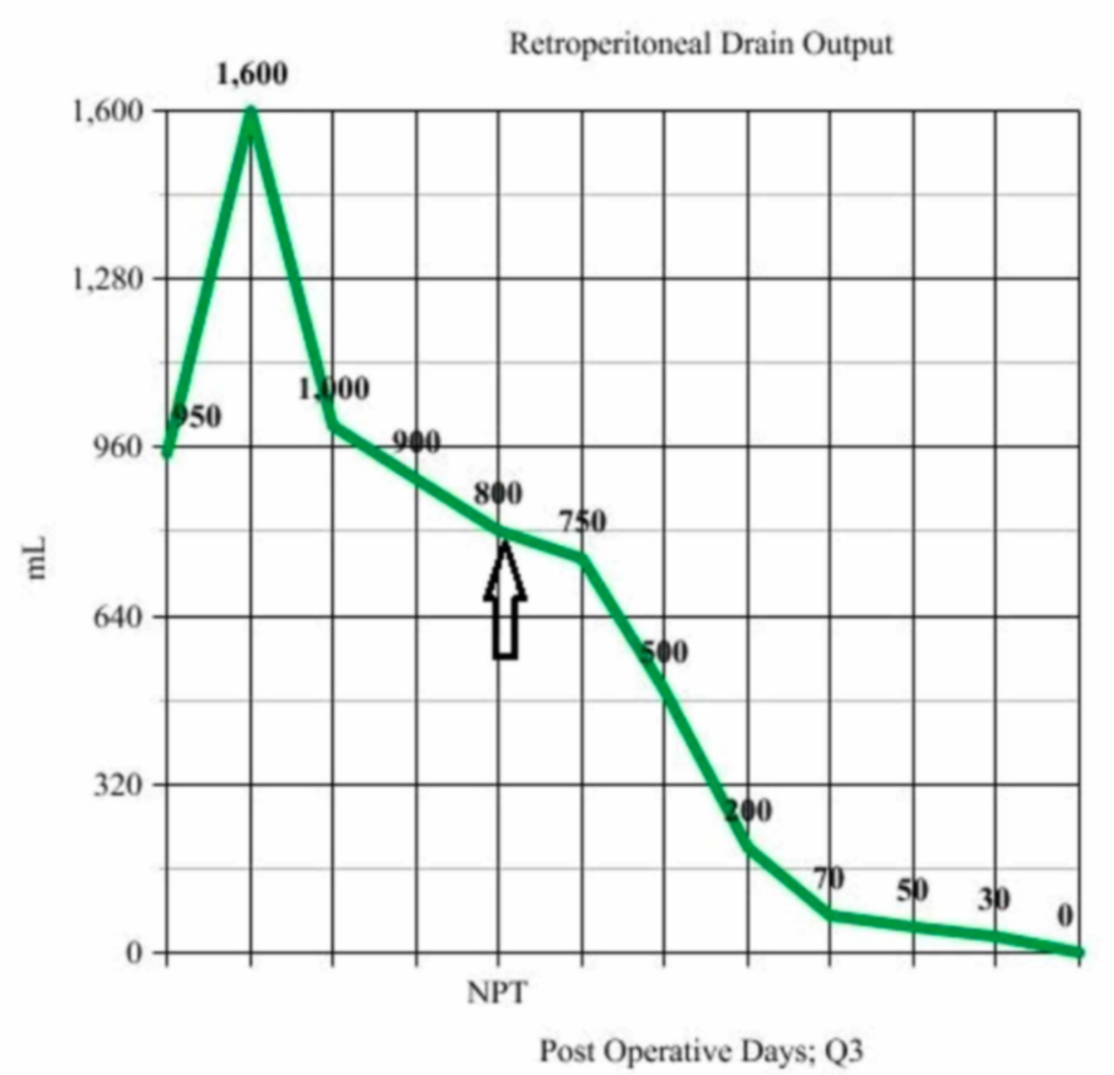
Daily retroperitoneal drain output averages every three days; Initiation of negative pressure therapy denoted by NPT

The application of negative pressure therapy made an immediate impact on the clinical status of the patient. It allowed the extension of negative pressure to the retroperitoneal space that was otherwise inadequately drained by the Jackson-Pratt drains. This was evidenced by her immediate cessation of fevers, improved diet tolerance, and progressive decrease in drain output volume, as referenced by Figure 5 below.

**Figure 6:**
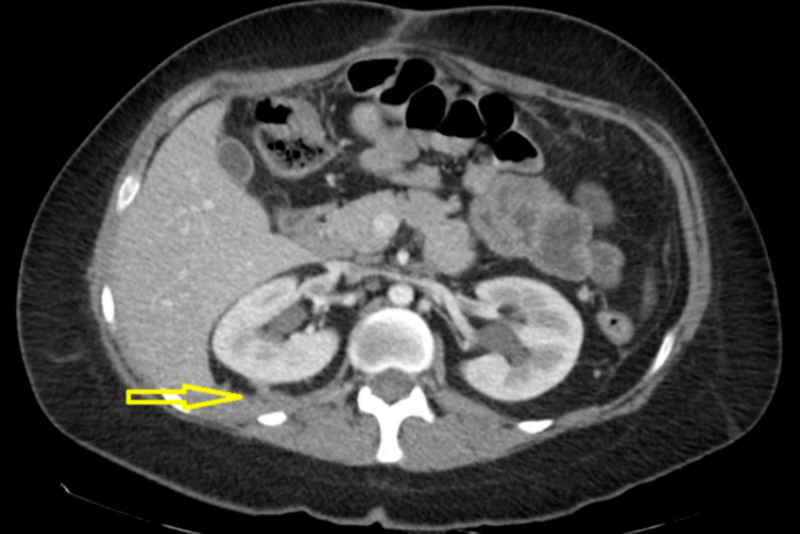
Axial image showing resolution of right pararenal fluid collections

After two weeks, an additional CT scan of the abdomen and pelvis was ordered to assess the progression of her retroperitoneal fluid collections. It revealed the resolution of the fluid collections in the right peri-colic gutter, right retroperitoneum, and near resolution of right peri-renal fluid collections (Figure 6).

The fistula completely closed, and drains were removed three weeks after the initiation of negative pressure therapy.

## Discussion

Non-operative management of a duodenal fistula consists of bowel rest, nasogastric decompression, proper drainage, and nutritional support. If the fistula fails to heal spontaneously, operative re-intervention is indicated but carries high mortality (30%) [2], especially in the setting of sepsis or obstruction. The retroperitoneal space is difficult to drain adequately, and fistulas in this location often involve a prolonged course, ranging on average 19-63 days [4].

More recently, the application of endo-luminal negative pressure for upper gastrointestinal leaks has been successfully applied in case studies involving iatrogenic duodenal perforation [3]. As it pertains to this case, an endo-luminal approach was not feasible due to the pyloric exclusion during her initial operation.

The application of negative pressure therapy via drains allowed access to the persistent retroperitoneal fluid collections that were inadequately drained by the Jackson-Pratt drains alone. Additionally, the retroperitoneal fluid collections were deemed inaccessible for additional drain placement via an interventional radiology approach. Previous case studies successfully used negative pressure wall suction for the management of duodenal fistulas, but this was through the application of wound vacuum through retroperitoneal open wound [4]. Extending negative pressure via drains to the retroperitoneal space allowed proper drainage, control of sepsis, and saved the patient additional surgical intervention. It also helped heal the excoriation of the skin around the drains, greatly improving local wound care. The continuous negative pressure therapy allowed more expedient closure of the fistula and led to a decrease in length of hospital stay.

This approach is of clinical interest as it allowed us to extend negative pressure intra-abdominally to the source of the problem. In a similar fashion, this technique could also be applied to cases of intra-abdominal and retroperitoneal sepsis, like infected collections from necrotizing pancreatitis and other gastrointestinal leaks. We anticipate that applying negative pressure therapy via drains can play a key role in the management of retroperitoneal infections and intestinal leaks.

## Conclusions

The closed incision negative pressure therapy via retroperitoneal drains greatly improved the clinical status of our patient, as evidenced by the cessation of sepsis, decreased fistula output, quicker closure of the fistula, and improved local wound care. The modality was well-tolerated by the patient and technically easy to perform. Within the limits of a case study, this article can serve as a reference for the management of duodenal fistula and other cases of intra-abdominal sepsis. Further case studies and research are needed to corroborate the application of this modality with improved outcomes in patients with duodenal fistula.
